# Supplementary material for: First complete mitogenomes of Diamesinae, Orthocladiinae, Prodiamesinae, Tanypodinae (Diptera: Chironomidae) and their implication in phylogenetics
Source: PeerJ. 2021 May 6;9:e11294. doi: 10.7717/peerj.11294 (PMC8106913; doi:10.7717/peerj.11294)
Supplement: Supplemental Information 1 [file peerj-09-11294-s001.doc]

**Supporting Information**

**Table S1. The best model for each partition of the five datasets.**

| Datasets | Partition names | Best Model |
| --- | --- | --- |
| PCG123 | CO1, CO2, CO3, CytB, ATP6, ND3; | GTR+I+G |
|  | ND2, ND6, ATP8; | TVM+I+G |
|  | ND5, ND1, ND4, ND4L. | TIM+I+G |
| PCG123R | CO1, CO2, CO3, CytB, ND3, ATP6; | GTR+I+G |
|  | ND2, ATP8, ND6; | TVM+I+G |
|  | ND5, ND1, ND4, ND4L; | TIM+I+G |
|  | 12S, 16S. | TVM+G |
| PCG12 | CO1, ATP6, ND3, CO2, CO3, CytB; | GTR+G |
|  | ND2, ND6, ATP8; | TVM+I+G |
|  | ND5, ND1, ND4, ND4L. | K81UF+G |
| PCG12R | CO1, ND3, ATP6, CO2, CO3, CytB | GTR+G |
|  | ND2, ND6, ATP8 | TVM+I+G |
|  | ND5, ND1, ND4, ND4L | K81UF+G |
|  | 12S, 16S. | TVM+G |
| AA | CO1, CO2, ATP6, CytB, CO3; | MTART+I+G |
|  | ND3, ATP8, ND6, ND2; | MTART+G+F |
|  | ND5, ND1, ND4, ND4L. | MTART+G+F |


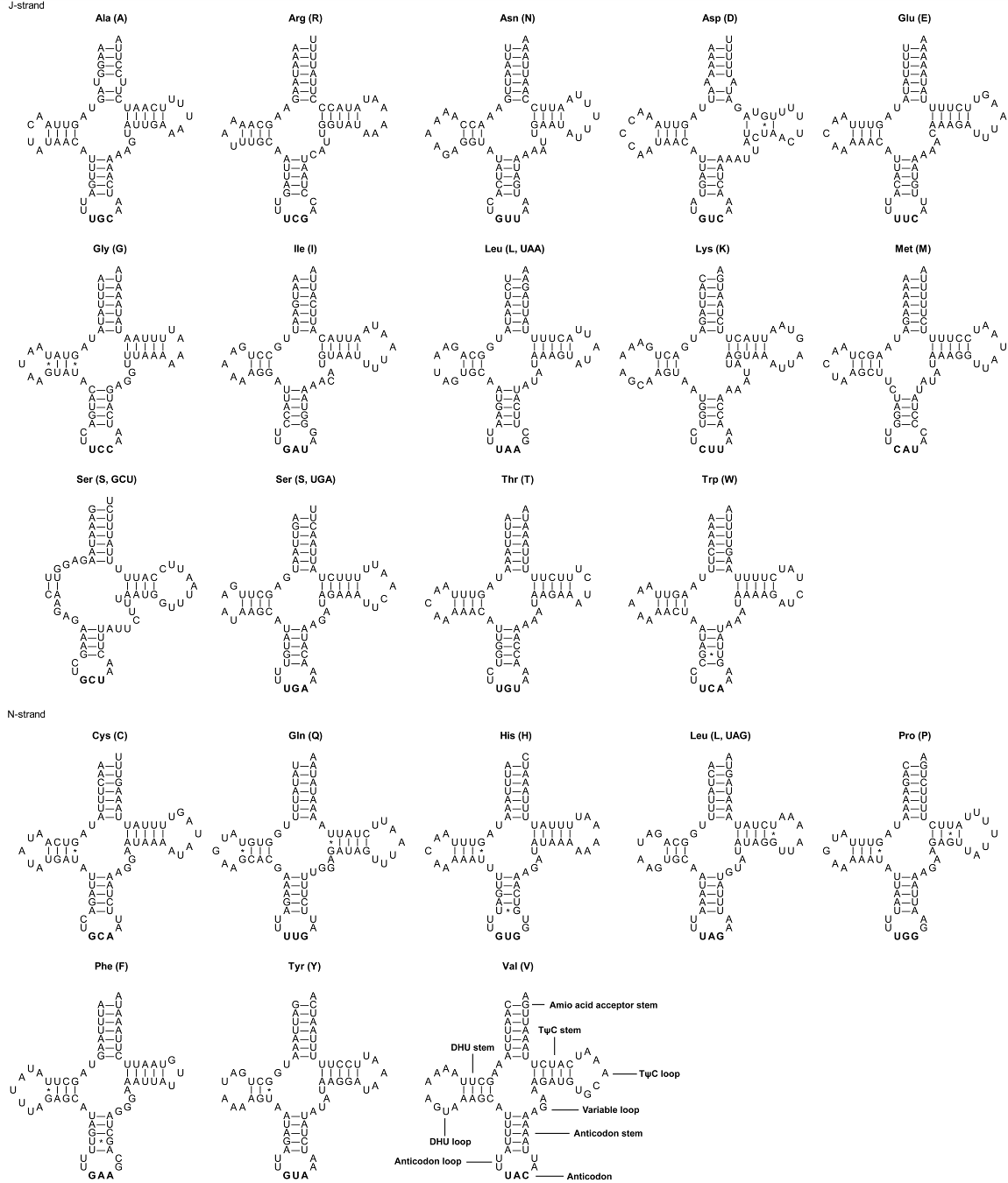


**Figure S1.** **Secondary structure of 22 tRNAs in *Chironomus tepperi.***


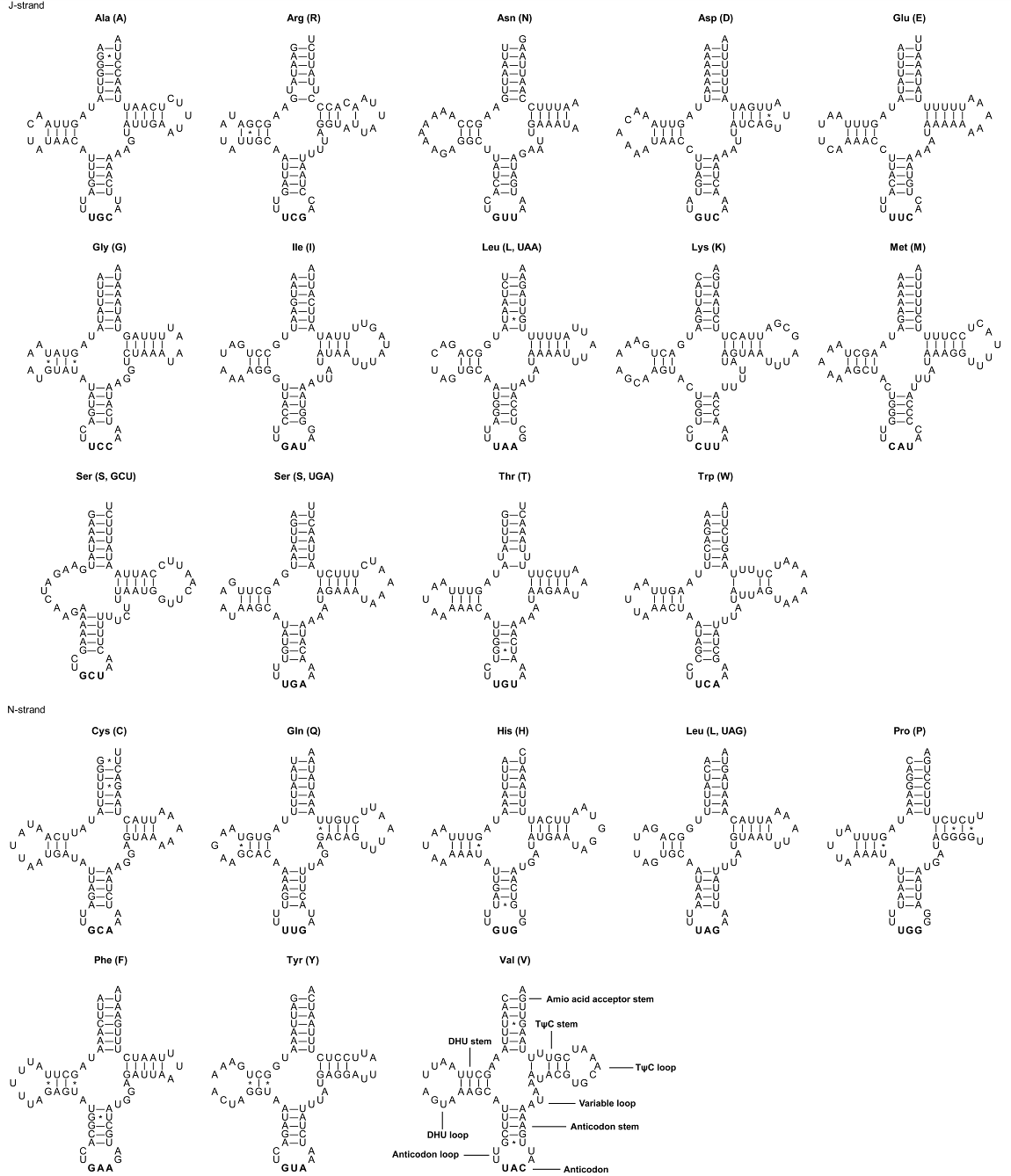


**Figure S2.** **Secondary structure of 22 tRNAs in *Potthastia* sp..**


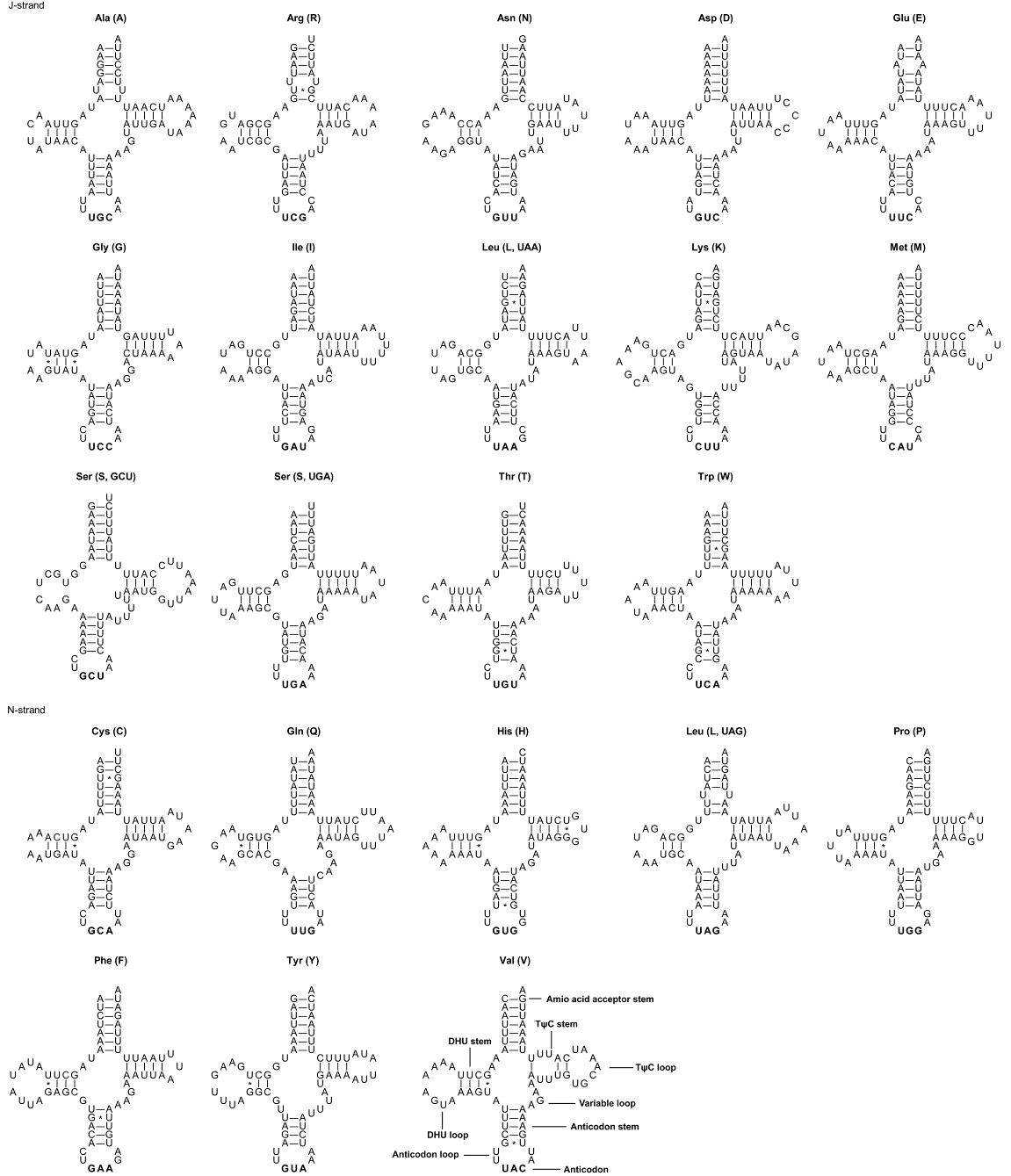


**Figure S3.** **Secondary structure of 22 tRNAs in *Rheocricotopus villiculus.***


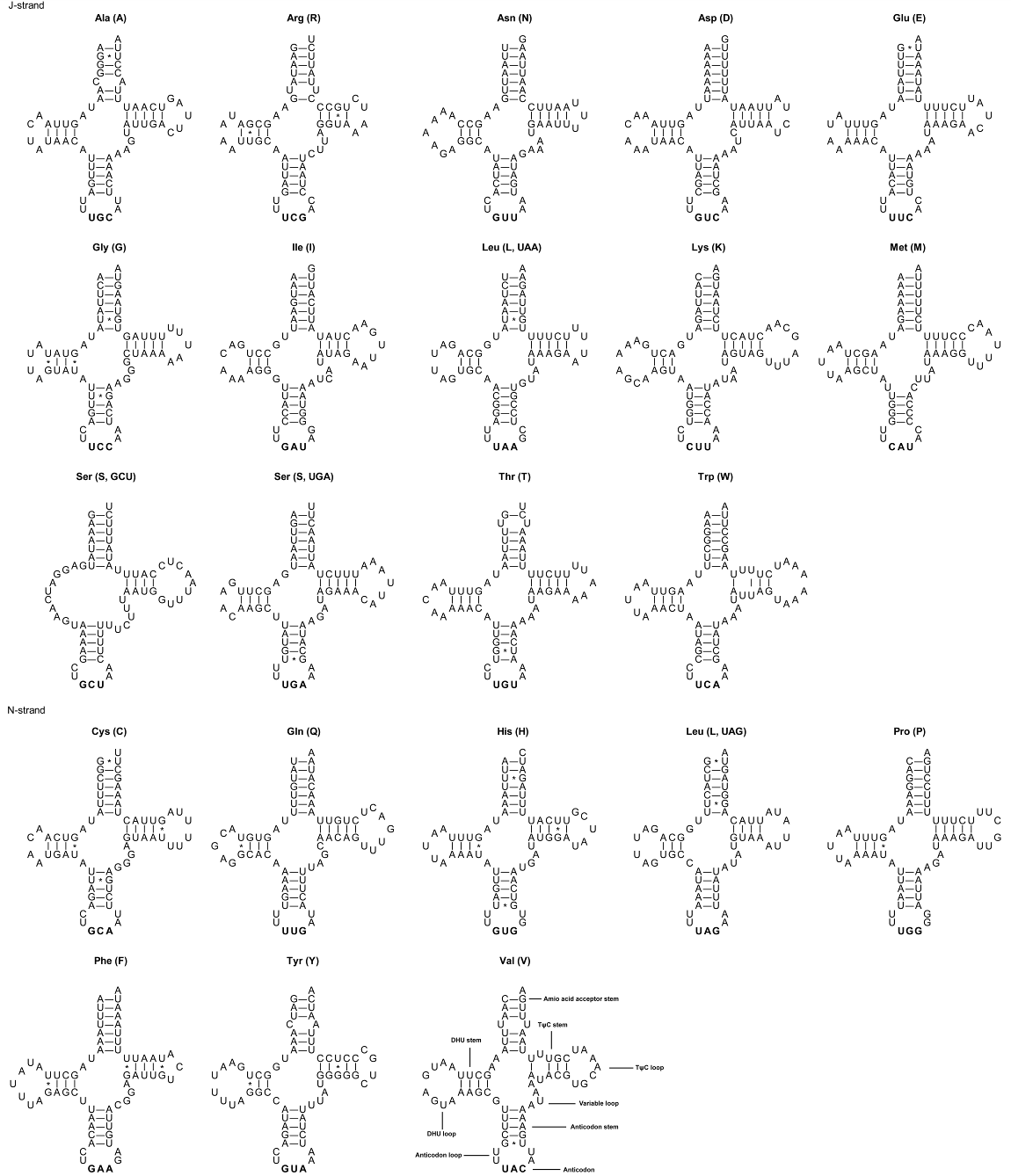


**Figure S4.** **Secondary structure of 22 tRNAs in *Parochlus steinenii.***


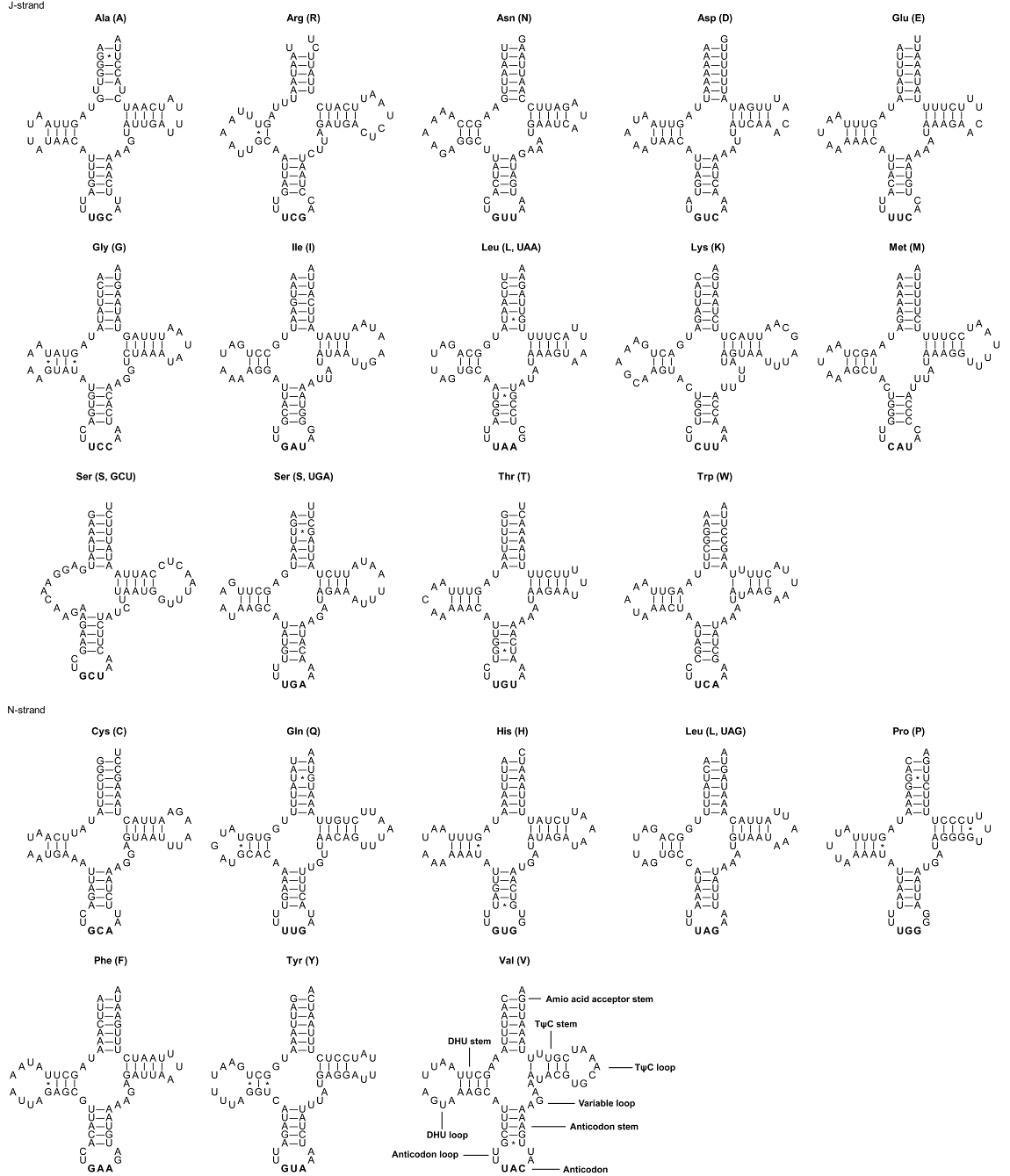


**Figure S5.** **Secondary structure of 22 tRNAs in *Prodiamesa olivacea.***


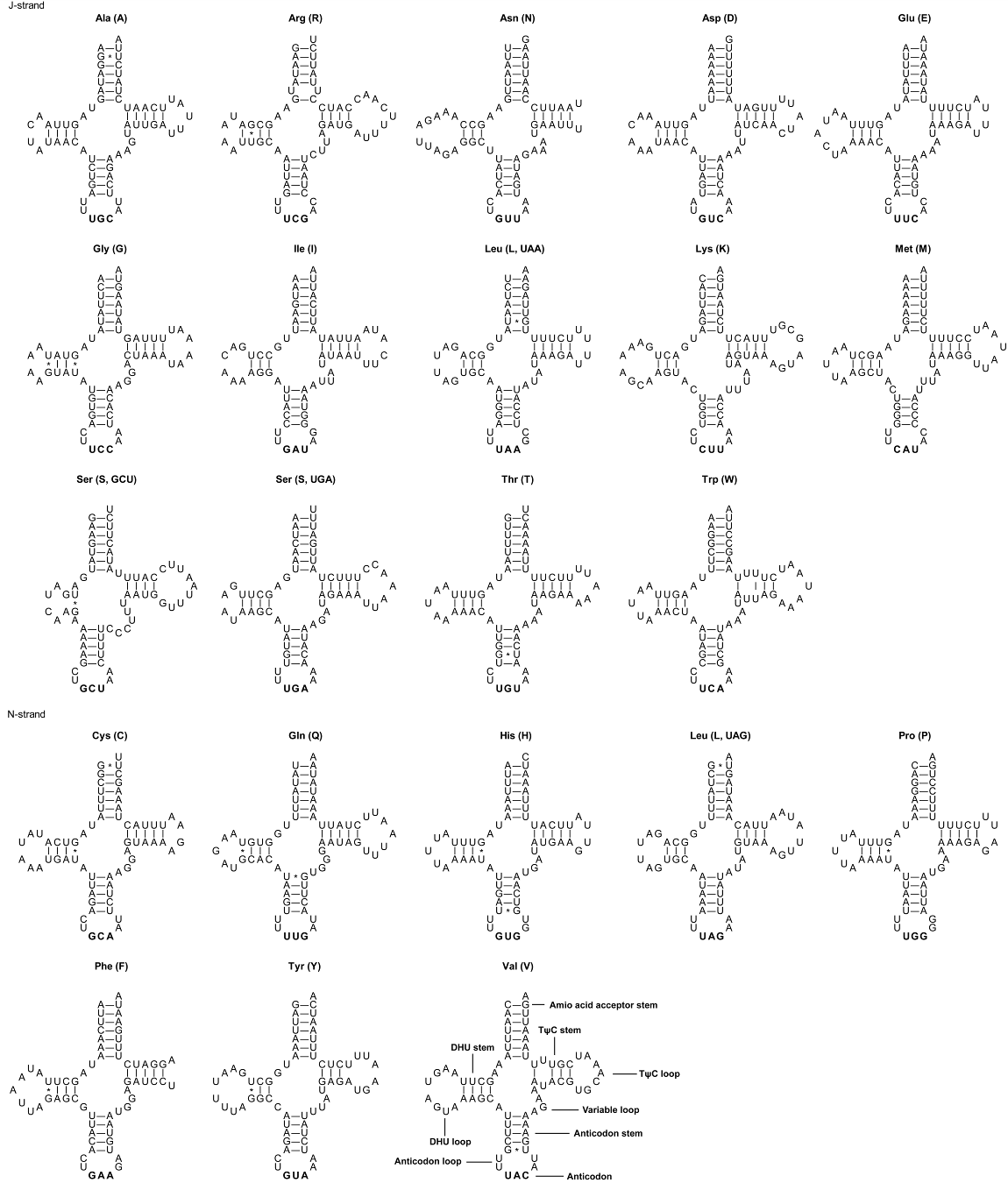


**Figure S6.** **Secondary structure of 22 tRNAs in *Clinotanypus yani.***


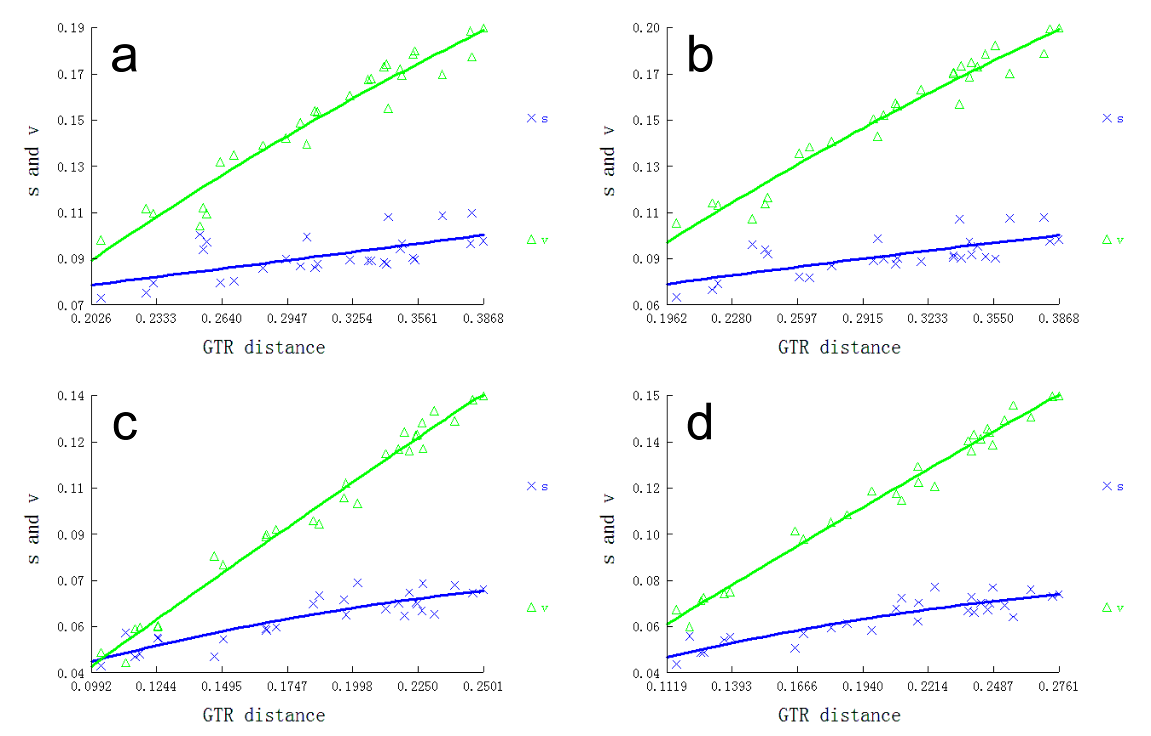


**Figure S7.** **Substitution patterns of the PCG123 (a), PCG123R (b), PCG12 (c), and PCG12R (d) datasets. The graphs represent the increase in GTR distance.**
